# Lipid composition and molecular species of phospholipid in oyster *Crassostrea lugubris* (Sowerby, 1871) from Lang Co Beach, Hue Province, Vietnam

**DOI:** 10.1002/fsn3.2385

**Published:** 2021-06-14

**Authors:** Thanh Tra Thi Le, Quoc Toan Tran, PeteVladimirovich Velansky, Tien Duc Dam, Long Giang Bach, Long Quoc Pham

**Affiliations:** ^1^ Institute of Natural Products Chemistry Vietnam Academy of Science and Technology Hanoi City Vietnam; ^2^ Graduate University of Science and Technology Vietnam Academy of Science and Technology Hanoi City Vietnam; ^3^ Department of Chemical Engineering Faculty of Chemistry and Environment Thuyloi University Hanoi City Vietnam; ^4^ A.V. Zhirmunsky Institute of Marine Biology of the Far Eastern Branch of the Russian Academy of Sciences Vladivostok Russia; ^5^ Institute of Marine Environment and Resources Vietnam Academy of Science and Technology Hanoi city Vietnam; ^6^ NTT Hi‐Tech Institute Nguyen Tat Thanh University Ho Chi Minh City Vietnam; ^7^ Center of Excellence for Biochemistry and Natural Products Nguyen Tat Thanh University Ho Chi Minh City Vietnam

**Keywords:** *Crassostrea lugubris*, fatty acids, high‐resolution mass spectrometry, lipid classes, molecular species, oyster, phosphatidylglycolic acid, phospholipids

## Abstract

Oysters are widely distributed worldwide, but are mainly concentrated in tropics and subtropics. Total lipid (TL), fatty acid (FA) composition of TL and polar lipid (PoL) fractions, phospholipid (PL) class, and molecular species composition in soft tissues of Crassostrea lugubris were investigated for the first time from Vietnam. Phosphatidylglycolic acid (PGA) is the new phospholipid class first identified in marine species in general and Crassostrea lugubris in particular. Main eight classes of PL were determined in PoL fraction: diphosphatidylglycerol (DPG), phosphatidylethanolamine (PE), phosphatidylcholine (PC), phosphatidylinositol (PI), phosphatidylserine (PS), ceramide aminoethylphosphonate (CAEP), CAEP with hydroxylated FAs (CAEP‐OH), and lysophosphatidylcholine. PE and PC accounted for approximately 63% of total known PL. Polyunsaturated FAs accounted for more than 30% of TL. Ninety molecular species of glycerophospholipids, including PGA, PE, PC, PS, PI, DPG, and PG, and sphingophosphonolipids (CAEP) were identified in PoL. Alkenyl‐acyl forms of glycerophospholipids were predominated in the molecular species of PGA, PE, and PS. PGA 38:1 (p18:0/20:1), PE 40:6 (p18:0/22:6 and p18:1/22:5), PC 30:0 (14:0/16:0), PS 38:1 (p18:0/20:1), PI 40:5 (20:1/20:4), PG 32:0 (16:0/16:0), DPG 88:24 (22:6/22:6/22:6/22:6), and CAEP 34:2 (d18:2/16:0) were major molecular species.

## Graphical abstract

This study presented a first structural characterization and quantification of phospholipid molecular species, in general, and phosphoglycolic acid, in particular, of Vietnamese oyster. Eight molecular species of PGA were found using HPLC‐HRMS. Other types of glycerophospholipid were identified, including PE, PC, PS, PI, DPG, and PG.

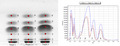

## 1 INTRODUCTION

Oysters have been recognized as one of the valuable and nutritious aquatic food. The significance of oysters lies in the abundance of important minerals such as zinc (158 ± 61 mg/100 g), calcium (203 ± 87 mg/100 g), magnesium (531 ± 65 mg/100 g), potassium (255 ± 51 mg/100 g), phosphorus (881 ± 189 mg/100 g), and iron (40 ± 11 mg/100 g) that are provided for the daily average diet of human (Abreu, 2020). The chemical composition and fatty acid profile of the tray‐cultured Pacific oyster (*Crassostrea gigas*) were measured and compared over a 13‐month period. The result showed that ranges for the chemical composition (dry flesh weight basis) were fat (7.8%–8.7%), protein (39.1%–53.1%), glycogen (21.6%–38.9%), and ash (4.0%–12.1%). In addition, the fatty acid profile remained relatively constant during 13 months of the study, with high concentrations of ɷ −3 fatty acids (18:3, 20:5, and 22:6) and low overall concentrations of saturated fatty acids (14:0, 16:0, and 18:0). They have been found to be responsible for a wide array of health benefits (Linehan, 1999).

Dietary supplements of PUFA are mainly in the forms of triacylglycerol (TAG), ethyl ester (EE), and phospholipid (PL) (Witte et al., 2014). These years, due to their better bioavailability, higher tissue‐delivery capacity, and enhanced health‐promoting effects, PUFAs in the PL form have been increasingly studied (Ghasemifard et al., 2014).

Individual separation of phospholipid molecular species using high‐performance liquid chromatography has been widely established (Blank et al., 1984; Patton et al., 1982). In mussel, for example, PC accounted for about 60% of the muscles liver and PL amounted to 53.8% of the sum of all PLs in the gills (Liu et al., 2012). In another study involving various cold‐water species, it was found that the PE content may contribute, on average, 24.3, 25.1, and 22.3% to muscles, liver, and gills, respectively (Velansky & Kostesky, 2008). PC, PE, PI, PS, CAEP, PG, and DPG were found, but sphingomyelin (SM) was not detected in the sea worms, mollusks, and arthropods (Kostesky & Velansky, 2009). In the Soft Coral *Xenia* sp., PC, PE, PE, CAEP, PI, and lysophosphatidylcholine (LPC) contributed 39.5, 20.8, 20.5, 9.7, 4.3, and 5.3% to the total phospho‐ and phosphonolipids, respectively (Imbs et al., 2015; Imbs et al., 2010). In polar lipids of *Meretrix lyrata*, a common edible marine clam, molecular species, including PE 36:1 (p18:0/18:1), PC 38:6 (16:0/22:6), PS 38:1 (p18:0/20:1), PI 40:5 (20:1/20:4), PG 32:0 (16:0/16:0), and CAEP 34:2 (16:2/d18:0) were prevalent among ninety‐eight identified species (Tran et al., 2019). In oyster species, identification of molecular species was widely reported. In the US pacific oysters, 19 molecular species of diacyl/alkylacyl/alkenylacyl PS and 22 diacyl/alkylacyl/alkenylacyl PE were identified (Chen et al., 2012). In the Pacific oyster *Crassostrea gigas,* PC diacyl +alkyl was the most abundant (33.7%), followed by PE (26.9%) and CAEP (14.5%) (Le Grand et al., 2011). Meanwhile, in Japanese oyster *Crassostrea gigas*, 19 and 16 molecular species of PE and PC were determined, respectively (Yeong et al., 1990). In other three marine bivalves, including *Pecten maximus*, *Crassostrea gigas,* and *Mytilus edulis*, phospholipid compositions were determined from their lipid extracts and four docosahexaenoyl chains (Do4DPG) were proved by NMR spectroscopy (Kraffe et al., 2002).

*Crassostrea lugubris (C. lugubris)* (Veneridae genus), also known as oysters, could be found in seashore and estuarine areas and is regarded as a valuable export product with high economic value (Sowerby, 1871). In addition, the oysters could serve as an efficient assimilator of nutrients, causing significant reduction in total nitrogen, total carbon and total phosphorous per hectare (Dame & Libes, 1993; Higgins et al., 2011; Hyun et al., 2013). Oysters are an economically and nutritionally important aquaculture species. As the data on their chemical composition in general and their lipid in particular are limited, our study aims to be the first to completely report the lipid of oyster *C. lugubris* collected in Lang Co Beach, Hue Province, located in the Central Coast region of Vietnam. The content of total lipid, fatty acids, lipid, and phospholipid classes was presented. We showed that the lipids from oysters contained six classes in which polar lipid occupied approximately a quarter of TL. Beside the molecular species such as PE, PC, PI, PS, CAEP, PG, and DPG in other research in oysters, mollusks, and marine animals, this research would be the first to identify new molecular species PGA with eight molecular species.

## 2 MATERIALS AND METHODS

### 2.1 Material

The oysters were collected in March 2018 in Lang Co Beach, Hue Province, Vietnam, and transferred to Institute of Natural Products Chemistry, Vietnam Academy of Science and Technology. Soft tissues of oysters were then separated and crushed.

GP standards including GPCho (18:0/18:1 diacyl, a16:0/18:1 alkyl‐acyl and p18:0/20:4 alkenyl‐acyl) were purchased from Avanti Polar Lipids, Inc.

Reagents including methanol, acetonitrile, chloroform, and ammonium formate were of HPLC grade and purchased from Merck Corp.

### 2.2 Lipid extraction

The crushed soft tissues were extracted for TL following a modified Bligh–Dyer extraction procedure (Bligh & Dye, 1959). For 10 g of oyster soft tissues, 30 ml of chloroform/methanol solution (1:2, v:v) was used to extract in 6 hr, at 30°C to afford the homogenate, which was then subjected to filtration to obtain the residue. The residue was then repeatedly extracted in chloroform (20 ml) in 6 hr at 30°C. Afterward, the obtained homogenates were pooled and added with 20 ml of H_2_O to separate the mixture into layers. After evaporating the lower layer, the TL was dissolved in chloroform. Total lipid was extracted with seven repetitions and stored at −5°C.

### 2.3 Lipid and phospholipid class analysis

The precoated silica gel plates (6 cm ×6 cm) Sorbfil PTLC‐AF‐V (Sorbfil, Krasnodar, Russia) was prepared to determine lipid class compositions. The plate was developed in two steps in which full length development using n‐hexane/diethyl ether/acetic acid (85:15:1, v:v:v) was performed first, followed by redevelopment with chloroform/methanol (2:1, v:v) for 5% length. Afterward, air‐drying was commenced over the plates, followed by spraying with 10% H_2_SO_4_ in methanol and heating at 240°C for 10 min (Imbs et al., 2015). These classes of TL were determined by comparison with standards.

Phospholipid class compositions were qualitatively analyzed by two‐dimensional thin‐layer chromatography (TLC) and quantitatively determined by one‐dimensional TLC. First, dissolution of the extracted lipids in chloroform (80 mg/ml) was performed, followed by spotting onto the one‐ and two‐dimensional TLC using the silica gel plates (10 cm ×10 cm). One‐dimensional TLC plates were developed with chloroform/methanol/28% aqueous ammonia/benzene ratio of 65:30:5:10 (v:v:v:v). Two‐dimensional TLC plates were developed in the first direction with chloroform/methanol/28% aqueous ammonia/benzene ratio of 65:30:5:10 (v:v:v:v). This chromatogram was dried for about 10 min and then developed in the second direction with chloroform/acetone/methanol/acetic acid/water ratio of 70:30:5:5:2 (v:v:v:v:v). Subsequently, one‐ and two‐dimensional TLC plates were air‐dried and sprayed with three solutions: ninhydrin, molybdate reagent, and 10% H_2_SO_4_ in methanol (Rouser et al., 1970; Skipski et al., 1964). In order to identify phospholipids on TLC plates, authentic standards and the specific spray reagents used earlier were employed.

Grayscale chromatograms were obtained using a flatbed scanner (Epson Perfection 2,400 PHOTO), and their band intensities were evaluated with software (Sorbfil TLC Video densitometer, Krasnodar, Russia) to determine the quantification of lipid classes.

### 2.4 Polar lipid separation

Polar lipid was separated by TLC plate preparation. Glass‐backed precoated ready‐made silica gel plates from Merck, Darmstadt F.RG., Cat. No. 5721, 20 × 20 cm × 0.25 mm from Macherey‐Nagel were used. All plates used were precleaned by running them in methanol: dichloromethane (2:1, v:v) and dried. The plates were activated before use by heating for 30 min in an oven at 130°C. They were dried for 10 min between migrations, in a vacuum desiccator at 250°C.

The one‐dimensional TLC separation technique was developed with chloroform/methanol/28% aqueous ammonia/benzene, 65:30:5:10 (v:v:v:v). Then, the plates were allowed to dry at room temperature.

After completion of TLC separation on Merck, the spots were located by phosphomolybdic acid and heated in an oven at 100–120°C for at least 2–5 min. The phosphomolybdic acid reagent composed of 5 g phosphomolybdic acid in 100 ml, ethanol plus 1 ml, and 70% perchloric acid (Pucsok et al., 1988).

### 2.5 Fatty acids analysis

Gas chromatography (GC) and gas chromatography–mass spectrometry (GC‐MS) equipment was used to analyze FAs. GC‐MS exactly provided the structure of fatty acids, while GC showed fatty acid contents. To perform GC analysis, a Shimadzu GC‐2010 chromatograph equipped with a flame ionization detector and a capillary column with dimensions of 30 m × 0.25 mm ×0.25μm (SUPELCOWAX 10, Supelco) was employed in conjunction with helium as the carrier gas (at 30 cm/s). The instruments used for performing GC‐MS analysis consisted of a gas chromatograph (Shimadzu GCMS‐QP5050A) (electron impact at 70 eV) equipped with a MDN‐5s (Supelco) capillary column (30 m × 0.25 mm ID) using helium as the carrier gas at 30 cm/s.

Lipid and polar lipid were first treated with 2% H_2_SO_4_ in methanol commenced in 2 hr at 80°C in a screw top vial, followed by purification by TLC development in hexane–diethyl ether (95:5, v:v). GC analysis was employed to analyze fatty acid methyl esters (FAME) with column temperature of 210°C. Identification of FA was carried out by comparing the obtained results with authentic standards and reported the equivalent chain lengths (Christie et al., 1988). Injector and detector temperatures were set at 240°C.

Fatty acids were structurally determined by performing GC‐MS against the corresponding FAME and subsequently, matching the obtained spectra with the NIST library and FA mass spectra archive (Harrabi et al., 2009; Mass spectrometry of Fatty Acid Derivatives, 2020). The thermal profile of the column was initiated at 160°C, followed by an acceleration at 2°C/min to 240°C that prolonged for 20 min. The injector temperature was set at 250°C.

### 2.6 Phosphatidylglycolic acid synthesis

The cabbage trans‐phospholipase D was used for phosphatidylglycolic acid (PGA) synthesis. Fresh cabbage tissue (1 g) was homogenized with an equal volume of distilled water by using IKA Ultra‐Turrax T25 homogenizer with S25N‐10G tool. The obtained homogenate was filtrated and centrifuged at 6,000 rpm for 10 min. The supernatant was used as the enzyme source. 10 mkg of PC (18:0/18:1 diacyl, a16:0/18:1 alkyl‐acyl and p18:0/20:4 alkenyl‐acyl [Avanti Polar Lipids]) in chloroform was evaporated to dryness in a standard 2‐ml vessel and sonicated with 100 mkl of Na‐glycolate buffer (2 M, pH 5.6 with 0.08 M CaCl_2_) for 10 min in an ultrasonic bath. Then, 100 mkl of cabbage extract and 20 mkl of hexane were added, and the mixture was allowed to react for 2 hr at room temperature. The reaction products were extracted by 300 mkl of chloroform and redissolved in 50 mkl of chloroform.

### 2.7 Phospholipid molecular species analysis

Phospholipid molecular species were identified and characterized by high‐performance liquid chromatography–high‐resolution mass spectrometry (HPLC–HRMS). The chromatograph was equipped with two LC‐20AD pump units, CTO‐20A column oven, SIL‐20A autosampler, CBM‐20A communications bus module, DGU‐20A5 degasser, and a Develosil 100–5 Si column (150 mm ×2 mm ID, 5 μm particle size) (Nomura Chemical).

Develosil was used in HILIC mode, and binary gradient was used to perform HPLC separation. The gradient included two solvents, A and B. The solvent A was acetonitrile/water (94:6, v:v) and B was pure water. Both solvents contained 20 mM acetic acid and 10 mM ammonia. The compositional progress of the gradient began at 0% of solvent B, which was then elevated to 20% over 30 min and kept for another 10 min. Afterward, the percentage was allowed to drop to 0% for 7 min. Cumulatively, the whole progress lasted 47 min. The flow rate was 0.2 ml/min. Detection of polar lipids was carried out by HRMS. Authentic standards were used to compare with results using Shimadzu LCMS Solution control and processing software (v.3.60.361). In each polar lipid class, the molecular species were individually quantified the peak areas of ion chromatograms (Boukhchina et al., 2004).

### 2.8 Mild acidic hydrolysis

This simple method was used for alkenyl forms of phospholipids determination. The sample was evaporated to dryness in a 2‐ml vial under a stream of argon. The vial was then inverted over five drops of HCl in a vial cap. After 4 min, the vial was immediately purged with argon for 5 min. Alkyl and acyl bonds are able to withstand this treatment, while alkenyl bond is not (Murphy et al., 1993). Samples were injected into HPLC‐HRMS to identify the ion chromatograms.

### 2.9 Statistical analysis

The difference between mean values was analyzed by one‐way analysis of variance (ANOVA), using Excel 2013 software, with seven repetitions. The results were presented as: mean ± *SD*.

## 3 RESULTS AND DISCUSSIONS

### 3.1 Lipid class composition

Total lipid was found in an amount of 2.54 ± 0.32% of wet weight of the oysters. Six classes of TL of the oysters were detected, including hydrocarbons and wax (HW), triacylglycerol (TAG), free fatty acids (FFAs), sterol (ST), polar lipid (PoL), and monoalkyl diacylglycerol (MADAG) (Table 1 and Figure S1 in supporting information). The qualitative composition was similar to that of other Bivalvia, zooplankton and coral which have been previously investigated (Abad et al., 1995; Imbs et al., 2010; Nelson et al., 2000; Tran et al., 2019). MADAG claimed a significant share of TL at 13.77%, which was in line with the lipid compositions of cnidarians and coral. Regarding PoL, this component only represented a relatively low content (22.46% of TL), in comparison with PoL in lipid compositions of *Cnidaria*, *Ctenophora,* and clam, which usually occupied more than half of TL.

**TABLE 1:** Main lipid classes (weight % of total lipid) of *C. lugubris*

No.	R_f_	Lipid class	Content (%)
1	0.02	Polar lipids	22.46 ± 1.38
2	0.08	Sterols	12.09 ± 0.84
3	0.20	Free fatty acids	5.65 ± 0.49
4	0.35	Triacylglycerols	34.95 ± 1.46
5	0.48	Monoalkyl diacylglycerols	13.77 ± 0.83
6	0.64	Hydrocarbons and waxes	11.07 ± 0.88

Other major lipid classes, including HW, ST, and TAG, accounted for 11.07, 12.09, and 34.95% of TL respectively. Those contents are in line with previous research on various oyster species (Abad et al., 1995; Imbs et al., 2015; Nelson et al., 2000; Tran et al., 2019). In particular, FFA content (5.65%) was lower than that of other oyster species, indicating that the collected samples were of high quality. The presence of sterols indicated the widespread of lipid class, which usually acts as membrane constituents, in marine species (Bernsdorff & Winter, 2003; Le Grand et al., 2011; Prato et al., 2010).

### 3.2 Phospholipid class composition

Resolving capability of two‐dimensional TLC technique against polar lipids has been demonstrated in previous studies. Furthermore, quantification of phospholipids by spot analysis and color development without prior elution from TLC adsorbent has been proved to be an efficient and quick routine (Medh & Weigel, 1989; Rouser et al., 1970). The main eight classes of phospholipid were determined on two‐dimensional thin layer in polar lipid of the *C. lugubris*, including diphosphatidylglycerol (DPG), phosphatidylethanolamine (PE), phosphatidylcholine (PC), phosphatidylinositol (PI), phosphatidylserine (PS), ceramide aminoethylphosphonate (CAEP), CAEP with hydroxy‐FAs (CAEP‐OH), and lysophosphatidylcholine (LPC) (Figure S2) The composition is similar to that of other previous studies (Lund & Chu, 2002; Yeong et al., 1990; Kraffe et al., 2002). On one‐dimensional thin layer, there were six identified classes of phospholipid. PS +CAEP‐OH and CAEP +PI did not separate on one way.

The composition of phospholipid classes was determined by one‐dimensional TLC (Table 2 and Figure S3). PE and PC are two main classes in phospholipid of oysters, accounting for 32.98 and 29.29% of total known phospholipids, respectively. This result is in agreement with previous studies (Le Grand et al., 2011; Lund & Chu, 2002; Yeong et al., 1990; Kraffe et al., 2002). LPC accounted for the lowest proportion with 3.72%. This lipid is usually a product of phospholipases A activity. LPCs are bioactive molecules that possess a large polar or charged head and a single hydrophobic carbon chain (Nieto‐Posadas et al., 2012). DPG, also known as cardiolipin (CL), exists in phospholipid composition in mammal with the content of about 10% (White, 1973). This is similar to the current DPG percentage of 9.32%. The PS +CAEP‐OH and CAEP +PI fractions accounted for 10.35 and 14.33% of total known phospholipids, respectively.

**TABLE 2:** Composition of main phospholipid classes (% of total phospholipid) of *C. lugubris*

No.	R_f_	Lipid class	Content (%)
1	0.07	Lysophosphatidylcholine (LPC)	3.72 ± 0.68
2	0.13	CAEP with hydroxy‐FAs (CAEP‐OH) and phosphatidylserine (PS)	10.35 ± 0.01
3	0.16	Ceramide aminoethylphosphonate (CAEP), phosphatidylinositol (PI)	14.33 ± 0.92
4	0.23	Phosphatidylcholine (PC)	32.98 ± 0.24
5	0.33	Phosphatidylethanolamine (PE)	29.29 ± 0.20
6	0.47	Diphosphatidylglycerol (DPG)	9.32 ± 0.49

### 3.3 Fatty acid composition

The fatty acid composition of *C. lugubris* comprised a total of 26 fatty acids whose carbon atom number ranged from 14 to 22 (Table 3). An abundant presence of FA was detected as 14:0, 16:0, 16:1*n*‐7, 18:0, 18:1*n*‐9, 18:1*n*‐7, 16:3*n*‐3, 20:4*n*‐6 (arachidonic acid AA), 20:5*n*‐3 (eicosapentaenoic acid EPA), and 22:6*n*‐3 (DHA). Saturated fatty acids occupied 35.82 and 31.53% in total and polar lipid FA contents, respectively. Monounsaturated fatty acids (MUFAs) accounted for 29.00 and 26.52% of unsaturated fatty acids (USFAs) in total and polar lipid, respectively. A major MUFA in the composition was C18 MUFA with the content of slightly higher than 7%. Polyunsaturated fatty acids (PUFAs) amounted to 71 and 73.48% of USFA in total and polar lipid, respectively. Among PUFA, EPA in polar lipid composition (8.86%) was lower by one half in comparison with that (16.38%) in total lipid composition. Likewise, the content of DHA was 10.56 and 12.28% in total and polar lipid composition, respectively. In addition, n‐3 PUFA amounted to 35.91% of total FA, approximately twelve times higher than the content of n‐6 PUFA, at 4.73% of total FA.

**TABLE 3:** Fatty acid composition (% of total and polar lipid) of *C. lugubris*

Fatty acids	Total lipids (%)	Polar lipids (%)
**SAFAs**	**35.82**	**31.53**
14:0	6.14 ± 0.30	2.48 ± 0.29
15:0	0.83 ± 0.03	0.84 ± 0.08
16:0	22.22 ± 0.35	17.78 ± 0.69
i−17:0	0.37 ± 0.02	1.32 ± 0.55
17:0	1.49 ± 0.07	1.79 ± 0.10
18:0	4.78 ± 0.50	7.32 ± 0.91
**MUFAs**	**17.60**	**13.31**
16:1*n*−7	5.41 ± 0.40	0.98 ± 0.53
18:1*n*−9	2.92 ± 0.16	2.09 ± 0.18
18:1*n*−7	6.38 ± 0.75	4.36 ± 0.13
20:1*n*−11	1.19 ± 0.26	3.11 ± 0.14
20:1*n*−9	0.15 ± 0.06	0.32 ± 0.06
20:1*n*−7	1.56 ± 0.29	2.46 ± 0.08
**PUFAs**	**43.08**	**36.88**
16:3*n*−3	3.18 ± 1.28	0.48 ± 0.15
18:2*n*−6	1.95 ± 0.12	1.31 ± 0.14
18:3*n*−3	1.68 ± 0.18	0.76 ± 0.11
18:4*n*−3	2.01 ± 0.28	0.67 ± 0.08
20:2 nmi	0.47 ± 0.08	0.25 ± 0.02
20:3*n*−6	0.16 ± 0.07	0.17 ± 0.02
20:4*n*−6	2.17 ± 0.20	3.60 ± 0.91
20:5*n*−3	16.38 ± 1.09	8.86 ± 0.91
21:3*n*−3	0.38 ± 0.09	1.21 ± 0.27
22:2 nmi	1.97 ± 0.51	5.32 ± 0.23
21:5*n*−3	0.69 ± 0.09	0.37 ± 0.06
22:5*n*−6	0.44 ± 0.11	0.87 ± 0.11
22:5*n*−3	1.04 ± 0.10	1.48 ± 0.12
22:6*n*−3	10.56 ± 0.88	12.28 ± 0.81
**DMAs**	**‐**	**12.41**
DMA 16:0	‐	0.20 ± 0.02
DMA 17:0	‐	0.42 ± 0.18
DMA 18:0	‐	11.54 ± 0.98
DMA 20:1	‐	0.26 ± 0.06
Other	3.50 ± 0.47	5.87 ± 0.74

Thus, the percentage of fatty acids in total lipids is mostly higher than that of polar lipids, possibly due to the presence of DMA and the choice of molecular structure of polar lipid classes. In the total lipid, the content of aldehyde dimethyl acetals (DMA) was absent, while DMA accounted for 12.41% of polar lipid composition, suggesting a possibility that plasmalogen PL may have produced long‐chain DMA under acidic hydrolysis polar lipids of oyster. Octadecanal dimethyl acetal (DMA 18:0) was the main DMA and accounted for 11.54% in polar lipid and 92.95% of detected DMA. In addition, in polar lipids, the content of long‐chain polyunsaturated fatty acids containing 21 or 22 carbon atoms increases. Specifically, their fatty acid content in total lipids and polar lipids are 14.39%/96.5% and 21.16%/81.72% of identified fatty acids, respectively.

### 3.4 HRMS of phosphatidylglycolic acid standards

**FIGURE 1:**
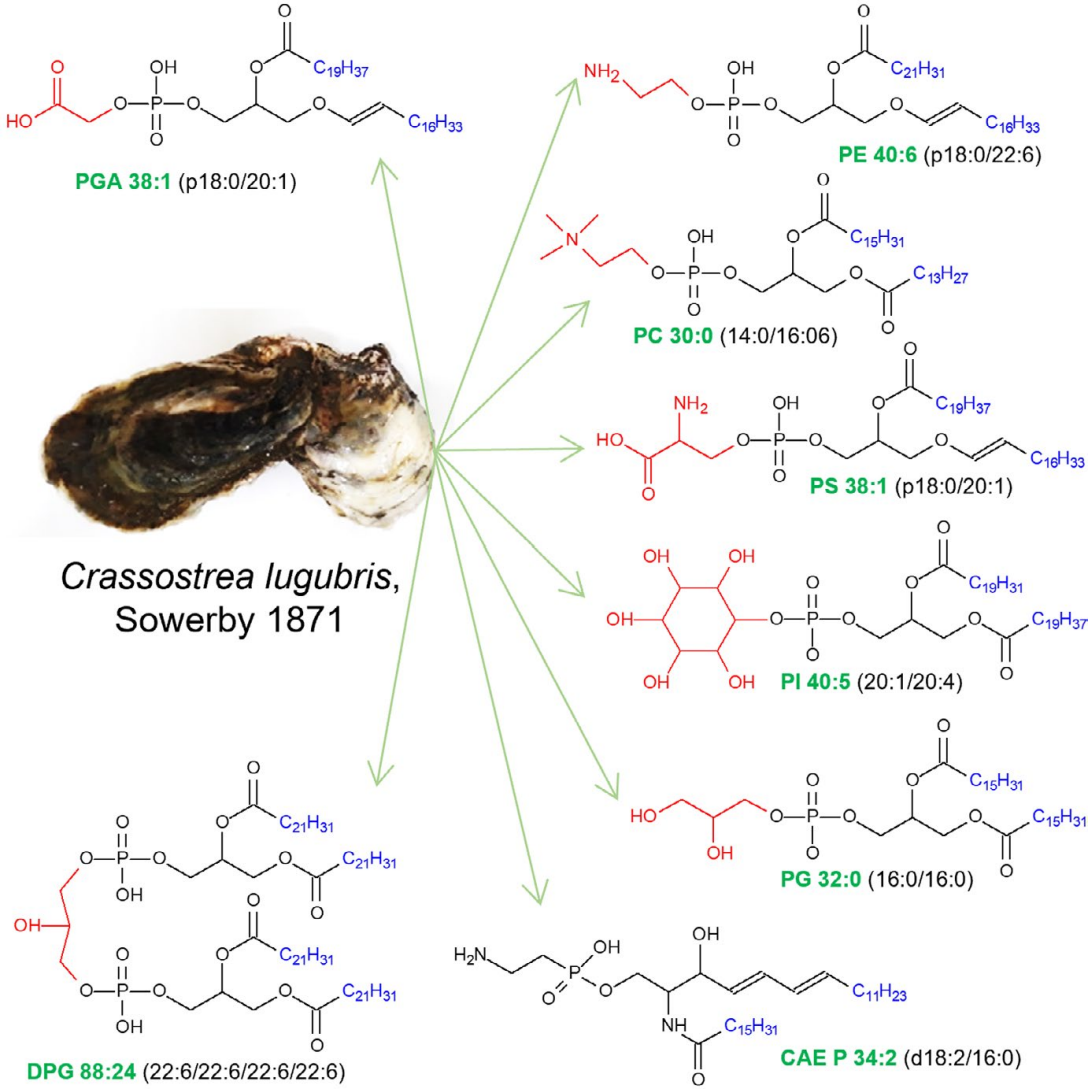
Supposed structural formula and mechanism of diacyl‐PGA fragmentation at (a) positive and (b) negative ionization

Fragmentation patterns of 1‐stearoyl‐2‐oleoyl‐*sn*‐glycero‐3‐phosphoglycolic acid PGA 36:1 (18:0/18:1) were analyzed by HRMS at ESI (±) conditions. PGA 36:1 (18:0/18:1) formed positive ammonium molecular ions [M+NH_4_]^+^ at *m*/*z* 778.5572, corresponding to composition [C_41_H_81_NO_10_P]^+^ (calculated 778.5593) and negative quasimolecular ions [M‐H]^‐^ at *m*/*z* 759.5171and [C_41_H_76_O_10_P]^‐^ (calculated 759.5182), respectively (Figure S4a,c). At the MS^2+^ stage, the ions at *m*/*z* 778.5572 eliminates ammonia, polar head, and glycolic acid moiety C_2_H_8_NO_6_P and formed ions at *m*/*z* 605.5388 (Figure S41b and Figure 1a). At the MS^2‐^ stage, the ions 759.5171 forms ion at *m*/*z* 701.5080 by elimination of glycolic acid C_2_H_2_O_2_ (cleavage 1 on Figure 1b). The ion at *m*/*z* 437.2594 was made by quasimolecular ion eliminated polar head and ketene 18:1 (cleavages 1 and 4 on Figure 1b). In addition, the ions at *m*/*z* 417.2372 and 419.2567 were formed by quasimolecular ion eliminated polar head and fatty acid 18:0 or 18:1 (cleavages 1 and 2 or 3 on Figure 1b). And ions with *m*/*z* 281.2518 ([C_18_H_33_O_2_]‐, calculated 281.2486) corresponding to 18:1 acid (oleic) anion, *m/z* 283.2621 ([C_18_H_35_O_2_]‐, calculated 283.2643), corresponding to 18:0 acid (stearic) anion (Figure S4d). Plasmalogen and alkyl‐acyl forms of PGA can be detected by fragments corresponding to [M‐H]^‐^loss R_2_ acid and glycolate (*m/z* 765.5076 ‐ *m/z* 403.2602 fragmentation on Figure S4e and *m/z* 717.5076 ‐ *m/z* 377.2429 fragmentation on Figure S4f). Only plasmalogen form was fragmented into alkenyl chain (*m/z* 765.5076 ‐ *m/z* 267.2693 fragmentation on Figure S4e). All detected ions of synthetic PGA are summarized in Table 4.

**TABLE 4:** Ions, formed by ionization and fragmentation of different molecular species of PGA

Ion^a^	Description	18:0/18:1	p18:0/20:4	a16:0/18:1
[M +NH_4_]^+^	Ammonium quasimolecular ion	778.5593	784.5487	736.5487
[M ‐C2H5O6P]^+^	Phosphoglycolate loss	605.5503	611.5398	563.5398
[M ‐H]^‐^	Dehydrogenated quasimolecular ion	759.5182	765.5076	717.5076
[M ‐C_2_H_2_O_2_]^‐^	Glycolate loss	701.5127	707.5021	659.5021
[R_1_‐COO]^‐^	R_1_ acyl chain fragment	283.2643	n/d	n/d
[R_2_‐COO]^‐^	R_2_ acyl chain fragment	281.2486	n/d	n/d
[R_1_‐CO]^‐^	R_1_ alkenyl chain fragment	n/d	267.2693	n/d
[M ‐R_1_COO ‐C_2_H_2_O_2_]^‐^	R_1_ acid & glycolate loss	417.2411	n/d	n/d
[M ‐R_2_COO ‐C_2_H_2_O_2_]^‐^	R_2_ acid & glycolate loss	419.2568	403.2618	377.2462
[M ‐R_1_CO ‐C_2_H_2_O_2_]^‐^	R_1_ ketene & glycolate loss	435.2517	n/d	n/d
[M ‐R_2_CO ‐C_2_H_2_O_2_]^‐^	R_2_ ketene & glycolate loss	437.2674	421.2724	395.2568

^a^ Theoretical calculated monoisotopic *m/z* values are given.

### 3.5 Phospholipid molecular species composition

#### 3.5.1 Molecular species of phosphatidylglycolic acid

This is the first new molecular species to be identified in PL of marine animal in general and oysters in particular. To determine the structure of molecular species of PGA from *C*. *lugubris*, the aforementioned data of the HRMS fragmentations of PGA standards (see Figure 1 and Table 4) were referenced. The quasimolecular anion [M‐H]^‐^ of each component lost polar head C_2_H_2_O_2_ at the MS^2‐^ stage. The subsequent fragmentation of components 2–6 formed the anions characterized acyl groups at the MS^2‐^ stage. Considering the fragmentation pathways and the monoisotopic molecular mass values, eight components, including four *O*‐alkenyl acylglycerolphosphatidylglycolic acid and four diacylglycerolphosphatidylglycolic acid (Table 5), were determined. Mild acidic treatment led to dissociation of alkenyl bonds, while diacyl PGA remains intact.

**TABLE 5:** Major molecular species composition of phosphatidylglycolic acid (PGA), phosphatidylethanolamine (PE), phosphatidylcholine (PC), phosphatidylserine (PS), phosphatidylinositol (PI), phosphatidylglycerol (PG), diphosphatidylglycerol (DPG), and ceramide aminoethylphosphonate (CAEP) from *C*. *lugubris*

Ion (*m/z*)	Symbol	Formula	Difference	% of each phospholipid class
**PGA ([M‐H]^‐^)**				
751.4948	16:0/20:5	C_41_H_69_O_10_P	0.0130	0.70
779.4895	18:0/20:5	C_43_H_73_O_10_P	0.0026	6.85
813.5612	18:0/22:2	C_45_H_83_O_10_P	0.0039	11.69
811.5487	18:1/22:2	C_45_H_81_O_10_P	0.0008	4.55
771.5526	p18:0/20:1	C_43_H_81_O_9_P	0.0019	35.05
763.4902	p18:0/20:5	C_43_H_73_O_9_P	0.0017	2.08
785.564	p19:0/20:1	C_44_H_83_O_9_P	0.0062	7.50
797.5695	p18:0/22:2	C_45_H_83_O_9_P	0.0007	24.97
**PE ([M‐H]^‐^)**				
750.5102	17:0/20:5	C_42_H_74_NO_8_P	0.0023	1.73
764.5220	18:0/20:5	C_43_H_76_NO_8_P	0.0016	7.06
762.5101	18:1/20:5	C_43_H_74_NO_8_P	0.0022	0.83
762.5040	18:1/20:5	C_43_H_74_NO_8_P	0.0039	3.19
796.5789	18:1/22:2	C_45_H_84_NO_8_P	0.0073	0.07
796.5838	18:1/22:2	C_45_H_84_NO_8_P	0.0024	0.17
788.5209	18:1/22:6	C_45_H_76_NO_8_P	0.0027	3.33
788.5193	18:1/22:6	C_45_H_76_NO_8_P	0.0043	3.33
756.5870	p18:0/20:1	C_43_H_84_NO_7_P	0.0043	6.4
748.5267	p18:0/20:5	C_43_H_76_NO_7_P	0.0020	10.37
762.5376	p18:0/21:5	C_44_H_78_NO_7_P	0.0067	2.36
782.6025	p18:0/22:2	C_45_H_86_NO_7_P	0.0044	17.37
776.5547	p18:0/22:5	C_45_H_80_NO_7_P	0.0053	6.47
774.5453	p18:0/22:6 or p18:1/22:5	C_45_H_78_NO_7_P	0.0010	27.21
796.6137	p19:0/22:2	C_45_H_84_NO_8_P	0.0108	0.86
788.5598	p19:0/22:6	C_46_H_80_NO_7_P	0.0002	2.47
800.5610	p20:1/22:6	C_47_H_80_NO_7_P	0.0010	1.03
**PC ([M+CH_3_COO]^‐^)**				
736.5198	14:0/14:0	C_36_H_72_NO_8_P	0.00639	7.26
764.5421	14:0/16:0	C_38_H_76_NO_8_P	0.00261	18.57
778.5534	15:0/16:0	C_39_H_78_NO_8_P	0.00696	5.76
792.5750	16:0/16:0	C_40_H_80_NO_8_P	0.00101	10.82
790.5604	14:0/18:1	C_40_H_78_NO_8_P	0.00004	6.77
810.5332	14:0/20:5	C_42_H_74_NO_8_P	0.00414	1.73
812.5442	16:0/18:4	C_42_H_76_NO_8_P	0.00051	3.96
816.5754	16:0/18:2	C_42_H_80_NO_8_P	0.00061	5.55
838.5616	16:0/20:5	C_44_H_78_NO_8_P	0.00124	13.41
844.6012	18:1/18:1	C_44_H_84_NO_8_P	0.00611	2.97
864.5726	16:0/22:6	C_46_H_80_NO_8_P	0.00341	0.81
750.5616	a16:0/14:0	C_38_H_78_NO_7_P	0.00384	5.75
824.5788	a16:0/20:5	C_44_H_80_NO_7_P	0.00229	3.32
850.5902	a16:0/22:6	C_46_H_82_NO_7_P	0.00654	4.83
878.6218	a18:0/22:6	C_48_H_86_NO_7_P	0.00627	8.5
**PS ([M‐H]^‐^)**				
808.5127	18:0/20:5	C_44_H_76_NO_10_P	0.00071	7.86
828.5701	n.d.	C_45_H_84_NO_10_P	0.00591	1.65
842.5864	18:0/22:2	C_46_H_86_NO_10_P	0.00526	9.82
842.5825	n.d.	C_46_H_86_NO_10_P	0.00916	1.25
854.5864	n.d.	C_47_H_86_NO_10_P	0.00526	1.86
800.5800	p18:0/20:1	C_44_H_84_NO_9_P	0.00109	35.04
814.5947	p19:0/20:1	C_45_H_86_NO_9_P	0.00146	8.11
826.5945	p18:0/22:2	C_46_H_86_NO_9_P	0.00224	30.53
818.5354	p18:0/22:6	C_46_H_78_NO_9_P	0.00126	3.88
**PI ([M‐H]^‐^)**				
835.5349	16:0/18:1	C_43_H_81_O_13_P	0.00070	14.85
855.4988	16:0/20:5	C_45_H_77_O_13_P	0.00410	4.68
869.5240	17:0/20:5	C_46_H_79_O_13_P	0.00545	2.43
885.5418	18:0/20:4	C_47_H_83_O_13_P	0.00805	2.38
883.5322	18:0/20:5	C_47_H_81_O_13_P	0.00200	15.36
861.5484	18:1/18:1	C_45_H_83_O_13_P	0.00145	2.38
881.5160	18:1/20:5	C_47_H_79_O_13_P	0.00255	4.36
917.6103	20:1/20:1	C_49_H_91_O_13_P	0.00215	0.92
911.5581	20:1/20:4	C_49_H_85_O_13_P	0.00740	28.36
909.5457	20:1/20:5	C_49_H_83_O_13_P	0.00415	16.22
925.5793	20:1/21:4	C_50_H_87_O_13_P	0.00185	0.21
935.5585	20:1/22:6	C_51_H_85_O_13_P	0.00700	7.85
**PG ([M‐H]^‐^)**				
721.4996	16:0/16:0	C_38_H_75_O_10_P	0.00291	99.57
767.4802	16:0/20:5	C_42_H_73_O_10_P	0.00666	0.43
**DPG ([M−2H]^2‐^)**				
806.4675	22:6/22:6/22:6/20:5	C_95_H_140_O_17_P_2_	0.00702	8.46
813.4734	22:6/22:6/22:6/21:5	C_96_H_142_O_17_P_2_	0.01087	6.44
819.4708	22:6/22:6/22:6/22:6	C_97_H_142_O_17_P_2_	0.01607	78.23
827.4734	22:6(OH)/22:6/22:6/22:6	C_97_H_142_O_18_P_2_	0.00579	6.88
**CAEP ([M‐H]^‐^)**				
615.4843	d16:1/16:0	C_34_H_69_N_2_O_5_P	0.0028	11.14
629.5020	d16:1/17:0	C_35_H_71_N_2_O_5_P	0.0008	0.92
629.5020	d17:1/16:0	C_35_H_71_N_2_O_5_P	0.0008	1.67
643.5162	d18:1/16:0	C_36_H_73_N_2_O_5_P	0.0022	6.81
641.5010	d18:2/16:0	C_36_H_71_N_2_O_5_P	0.0018	17.11
639.4854	d18:3/16:0	C_36_H_69_N_2_O_5_P	0.0017	11.82
657.5259	n.d.	C_37_H_75_N_2_O_5_P	0.0082	1.63
655.5123	d19:2/16:0	C_37_H_73_N_2_O_5_P	0.0061	2.91
655.5123	n.d.	C_37_H_73_N_2_O_5_P	0.0061	0.52
653.5021	d19:3/16:0	C_37_H_71_N_2_O_5_P	0.0007	9.43
669.5290	d20:2/16:0 + d18:2/18:0	C_38_H_75_N_2_O_5_P	0.0051	4.06
667.5126	d19:3/17:0	C_38_H_73_N_2_O_5_P	0.0058	3.48
667.5117	n.d.	C_38_H_73_N_2_O_5_P	0.0067	1.45
681.5300	d19:3/18:0	C_39_H_75_N_2_O_5_P	0.0041	1.84
681.5275	n.d.	C_39_H_75_N_2_O_5_P	0.0066	0.48
631.4855	n.d.	C_34_H_69_N_2_O_6_P	0.0035	0.48
657.4993	t18:2/16:0	C_36_H_71_N_2_O_6_P	0.0016	5.04
655.4831	d18:3/16:0(OH)	C_36_H_69_N_2_O_6_P	0.0011	4.44
669.4956	t19:3/16:0	C_37_H_71_N_2_O_6_P	0.0021	0.61
669.4963	d19:3/16:0(OH)	C_37_H_71_N_2_O_6_P	0.0014	10.96
685.5234	n.d.	C_38_H_75_N_2_O_6_P	0.0056	1.75
683.5097	n.d.	C_38_H_73_N_2_O_6_P	0.0037	0.12
697.5234	t19:3/18:0	C_39_H_75_N_2_O_6_P	0.0056	1.33

Compound PGA p38:1 was an *O*‐alkenyl acylglycerolphosphatidylglycolic acid, measured *m/z* 771.5553 and calculated *m/z* 771.5540 for [M‐H]^‐^ ion. On the MS^2‐^, four fragments were formed by quasimolecular anion lost polar head C_2_H_2_O_2_ (*m/z* 713.5369), lost polar head and fatty acid 20:1 (*m/z* 403.2611) and lost polar head and ketene 20:1 (*m/z* 421.2712). The *m/z* 267.2684 [C_18_H_35_O] ^‐^ was formed by alkenyl 18:0 group (Figures S5, S6).

#### 3.5.2 Molecular species of phosphatidylethanolamine

In the marine oysters, PE included alkenyl‐acyl and diacyl forms, but not alkyl‐acyl form (Chen et al., 2012). Among PE from *C. lugubris*, nineteen components were identified and alkenyl acyl glycerophosphoethanolamine (ethanolamine plasmalogen PlsEtn) was abundantly found in the PE composition, accounting for 74.56% of total PE species (Table 5). Owing to the alkenyl linkages at the *sn‐*1 position, the liberation of the fatty acid (FA) anion ([RCOO]^–^) can only occur from the *sn‐*2 position (Tran et al., 2019). Nine alkenyl‐acyl forms were determined with *sn*‐1plasmenyl linkages, such as p18:0, p18:1, p19:0, and p20:0. Among them, phosphatidyl p18:0/22:6; p18:0/22:2; p18:0/20:5; p18:0/20:1; and p18:0/22:5 were the predominant species (Table 5).

Total diacyl forms only accounted at 24.44% with some main species 18:0/20:5; 18:1/20:5, and 18:1/22:6 (Table 5).

To determine the structure of molecular species of PE, PC, PS, PI, and CAEP, we applied the data of the HRMS fragmentations of previously described PE standards (Chen et al., 2012; Imbs et al., 2015; Kraffe et al., 2002). HRMS spectra of all components of formula species of PE (Figure S7) exhibited signals of positive quasimolecular ions [M + H]^+^, cluster ions [M + H+C_2_H_8_NO_4_P]^+^ and negative quasimolecular ions [M‐H]^‐^ (Figure S7). For PE p40:2, signals of negative quasimolecular ions [M‐H]^‐^ at *m*/*z* 782.6025 ([C_45_H_85_NO_7_P]^‐^, calculated 782.6069, difference 0.0044), positive quasimolecular ions [M + H]^+^ at *m*/*z* 784.6164 ([C_45_H_87_NO_7_P]^+^, calculated 784.6215, difference 0.0051), and positive fragment ions [M + H ‐ C_2_H_8_NO_4_P]^+^ at *m*/*z* 643.5985 ([C_43_H_79_O_3_]^+^, calculated 643.6024, difference 0.0044) were observed (Figure S7).

In the MS^2‐^ spectrum of the parent ion [M‐H]^‐^ of PE p40:2, strong signal from acyl 22:2 fragment (*m/z* 335.2977), weak signals of 22:2 acyl (446.3023) and ketene (464.3130) losses, and alkenyl 18:0 (268.3561) fragment were observed.

#### 3.5.3 Molecular species of phosphatidylcholine

The results in *C. lugubris* were similar to hard clams. Different to PE, the percentage of diacylglycerophosphatidylcholine was about 3.5 times as abundant as PakCho in total PC, at 77.60% in comparison with 22.40%. In total, 15 PC components were determined (Table 5).

Mass spectrometry spectra of all PC molecular species showed signals of positive quasimolecular ions [M + H]^+^, negative acetate molecular ions [M+CH_3_COO]^‐^ and cluster ions [M‐CH_3_]^‐^. Acetate molecular ions ([M+CH_3_COO]^‐^) of each component lost methyl acetate at the MS^2‐^ stage (Figure S8). The component having highest percentage in PC, PC 30:0, for instance, formed negative acetylated molecular ion [M +CH_3_COO] ^‐^ at *m*/*z* 764.5421 ([C_40_H_79_NO_10_P]^‐^, calculated 764.5447, difference 0.0026), positive quasimolecular ion [M + H]^+^ at *m*/*z* 706.5415 ([C_38_H_79_NO_8_P]^+^, calculated 706.5381, difference 0.0034), and fragment ion [M‐CH_3_]^‐^ at 690.5136 ([C_37_H_73_NO_8_P]^‐^, calculated 690.5074, difference 0.0057) (Figure S8).

At the MS^2‐^ stage, a molecule of C_3_H_6_O_2_ (methyl acetate) was eliminated by the ions at *m*/*z* 764.5421 and ions were formed at *m*/*z* 690.5136, which indicated the formation process of the quasimolecular negative ions by the addition of acetate ion to the lipid molecule. On MS^2‐^ of component lost methyl acetate, two carboxylate anions of 14:0 at *m*/*z* 227.2013 (calculated 227.2017, different 0.0003) and 16:0 at *m/z* 255.2326 (calculated 255.2330, different 0.0005) were observed (Figure S8). Normally, the *sn*‐2 of PL is the preferred position for PUFAs (Tran et al., 2019). Therefore, PC 30:0 was characterized as diacylglycerophosphocholine 14:0/16:0.

#### 3.5.4 Molecular species of phosphatidylserine

We detected 9 components (Table 5). It is shown that alkenyl‐acyl glycerophosphoserine majorly constituted PS profile, which is similar to that of PE. The four components of alkenyl‐acyl glycerophosphoserine amounted to 82.32% of total PS species.

The signal of negative quasimolecular ions [M‐H]^‐^ was found in all components of formula species of PS and no formation of positive quasimolecular ions was observed. In each component, the MS^2‐^ spectrum of [M–H]^‐^exhibited a signal of characteristic ion [M–H–C_3_H_5_NO_2_],^‐^indicating the absence of serine group (Figure S9). This fragmentation is different from those of negative quasimolecular ions of PE. Negative quasimolecular ion [M‐H]^‐^, for instance, was formed by PS 38:1, at 800.5800 ([C_44_H_83_NO_9_P]^‐^, calculated 800.5805, difference 0.00099). On MS^2‐^, the loss of serine (713.5410), serine and acyl (403.2610), H_2_O, serine and acyl groups (421.2729) was observed. However, fragments, corresponding to fatty acids was not detected (Figure S8). The component PS 38:1 was determined as alkenyl‐acyl glycerophosphoserine, p18:0/20:1.

#### 3.5.5 Molecular species of phosphatidylinositol

Among PI of oysters, we determined 12 components (Table 5). In addition, alkenyl acyl glycerophosphoinositol was absent in PI. All ten components PI were diacylglycerophosphoinositol with fatty acids 16:0, 18:0, 18:1, 20:1, 20:4, 20:5, 22:6, 21:4.

As suggested by results of PI molecular species, negative quasimolecular ions [M‐H]^‐^ seem to have formed. Furthermore, several characteristic ions such as PI 40:5 were also found, according to the MS^2‐^ fragmentation of the ions [M‐H]^‐^ of PI (Figure S10). The formula for fragmentation 911.5581 was [C_49_H_84_O_13_P]^‐^, calculated 911.5650 (difference 0.0074). Simultaneous loss of inositol, acyl groups and carboxylate anion of fatty acid has been detected in MS^2‐^ spectra signal of quasimolecular ion of component PI 40:5. In addition, quasimolecular ion has been also found to lose diacyl groups, as reflected by fragmentation 297.0307. This was the important fragmentation to determine molecular species of PI. The component PI 40:5 was determined as diacylglycerophosphoinositol 20:1/20:4 from acyl fragment 20:1 (*m/z* 309.2787).

#### 3.5.6 Molecular species of phosphatidylglycerol

We determined 2 components, which were PG 32:0, 36:5 constituting phosphatidylglycerol (PG) (Table 5).

Signals indicating the negative quasimolecular ion 721.4983 ([C_38_H_74_O_10_P]^‐^, calculated 721.5020, difference 0.00501) was found in the MS^2‐^ spectra of component PG 32:0. The spectra also suggested high intensity carboxylate anion of 16:0 FA (255.2314) and low intensity fragments according to acyl loss (465.2599) and glycerol and acyl loss (391.2268) (Figure S11). As a result, this compound was determined as diacylglycerophosphoglycerol 16:0/16:0.

#### 3.5.7 Molecular species of diphosphatidylglycerol

Structural assignment could be elaborated by the MS^2‐^spectra of the [M‐2H]^2‐^ ions obtained with HPLC‐HRMS (Hsu et al., 2005). We found 4 components (Table 5), amounting to over 94% of FA 22:6*n*‐3, which is similar to previous study in some marine bivalves (Kraffe et al., 2002).

Double negative quasimolecular ion 819.4708 ([C_97_H_140_O_17_P_2_]^2‐^, calculated 819.4788, difference 0.0161) was found in the spectra of component DPG 88:24. In addition fragmentations of carboxylate anion of fatty acid 22:6 (327.2312) and hydrocarbon from FA 22:6 lost carboxylic group (283.2429) was found. The component DPG 88:24 (Figure S12) was determined as tetraacyldiglycerophosphoglycerol 22:6/22:6/22:6/22:6.

#### 3.5.8 Molecular species of ceramide aminoethylphosphonate

Corresponding to detected mass spectra, fifteen CAEP molecular species were identified (Table 5). Furthermore, signals corresponding to negative quasimolecular ions [M‐H]^‐^, positive quasimolecular ions [M + H]^+^ and positive cluster‐molecular ions [M+Na]^+^, [M + H‐H_2_O]^+^,[M + H ‐polar head]^+^,[M + H ‐H_2_O ‐polar head]^+^ were also found.

Several notable signals included signals of negative quasimolecular ions [M‐H]^‐^, at *m*/*z* 641.4997 ([C_36_H_70_N_2_O_5_P]^‐^, calculated 641.5022, difference 0.00308), positive quasimolecular ions [M + H]^+^ at *m*/*z* 643.5150([C_36_H_72_N_2_O_5_P]^+^, calculated 643.5179, difference 0.00306), positive cluster‐molecular ions [M+Na]^+^ at *m*/*z* 665.4967 ([C_36_H_71_N_2_O_5_PNa]^+^, calculated 665.4998, difference 0.00082), [M + H‐H_2_O]^+^ at *m/z* 625.5056 ([C_36_H_70_N_2_O_4_P]^+^, calculated 625.5073, difference 0.00117),[M + H‐polar head]^+^ at *m/z* 518.4890 ([C_34_H_64_NO_2_]^+^, calculated 518.4937, difference 0.00416), and [M + H‐H_2_O‐polar head]^+^ at *m/*z 500.4755 ([C_34_H_62_NO]^+^, calculated 500.4831, difference 0.00709) of CAEP 34:2 (Figure S13).

It was found from MS^2‐^ spectrum of [M‐H]^‐^ of CAEP that the sphingolipid represented the fragmentation pattern that is different from that of negative quasimolecular ions of glycerophospholipids (Figure S13). The MS^2‐^ spectra also suggested the presence of four ions, which is indicative of long‐chain 18:2 base and 16:0 fatty acid group.

## 4 CONCLUSIONS

The present study analyzed lipid composition of *C. lugubris* harvested from Lang Co Beach, Hue Province, Vietnam. The total lipid accounted for 2.54 ± 0.32% of wet weight of the oyster soft tissues. Six lipid classes consisting of HW, TAG, FFA, ST, PoL, and MADAG were detected. Among these classes, PoL and TAG claimed the largest share of TL, at 57.41%. In addition, one‐ and two‐dimensional TLC revealed main eight classes of phospholipid, including DPG, PE, PC, PI, PS, CAEP, CAEP‐OH, and LPC. The large proportion of FA such as EPA, DHA, AA, and DPA at over 30% of total lipid proved high nutrient and economic value of oysters.

This study presented a first structural characterization and quantification of phospholipid molecular species, in general, and phosphoglycolic acid, in particular, of Vietnamese oysters. Eight molecular species of PGA were found by using HPLC‐HRMS. Other types of glycerophospholipid were identified, including PE, PC, PS, PI, DPG, and PG. One type sphingophosphonolipid was identified to be CAEP. Eighty‐three molecular species were identified in polar lipids of the oysters. Alkenyl acyl forms of glycerophospholipids predominated in the molecular species determined. PGA 38:1 (p18:0/20:1), PE 40:6 (p18:0/22:6 and p18:1/22:5), PC 30:0 (14:0/16:0), PS 38:1 (p18:0/20:1), PI 40:5 (20:1/20:4), PG 32:0 (16:0/16:0), DPG 88:24 (22:6/22:6/22:6/22:6), and CAEP 32:2 (16:2/d18:0) were the major molecular species.

## CONFLICT OF INTEREST

The authors declare no conflict of interest.

## ETHICAL APPROVAL

The study's protocols and procedures were ethically reviewed and approved by Vietnam Academy of Science and Technology, Hanoi City, Vietnam.

## Supporting information

App S1
