# Supplementary material for: Lipid composition and molecular species of phospholipid in oyster Crassostrea lugubris (Sowerby, 1871) from Lang Co Beach, Hue Province, Vietnam
Source: Food Sci Nutr. 2021 Jun 14;9(8):4199–210. doi: 10.1002/fsn3.2385 (PMC8358366; doi:10.1002/fsn3.2385)
Supplement: Supplementary file 1 — App S1 [file FSN3-9-4199-s001.docx]

**Appendix S1**

**Supporting information**

| 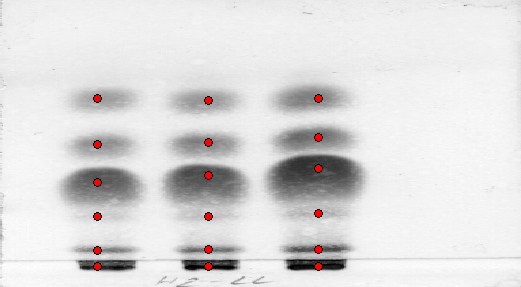  Track 1  Track 2  Track 3  1  2  3  4  5  6  1  2  3  4  5  6  1  2  3  4  5  6 | 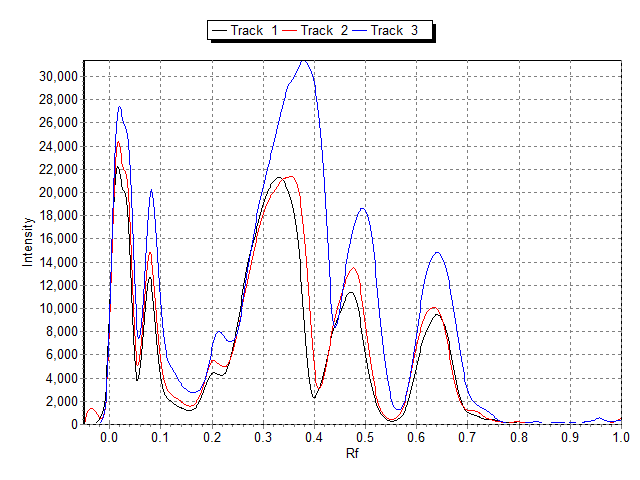  1  2  3  4  5  6 |
| --- | --- |

**Figure S1.** Sorbfil TLC determination of lipid classes of *C. lugubris*. 1 – polar lipids (PoL), 2 – sterols (ST), 3 – free fatty acids (FFA), 4 – triacylglycerols (TAG), 5 – monoalkyl diacylglycerols (MADAG), 6 – hydrocarbons and waxes (HW).


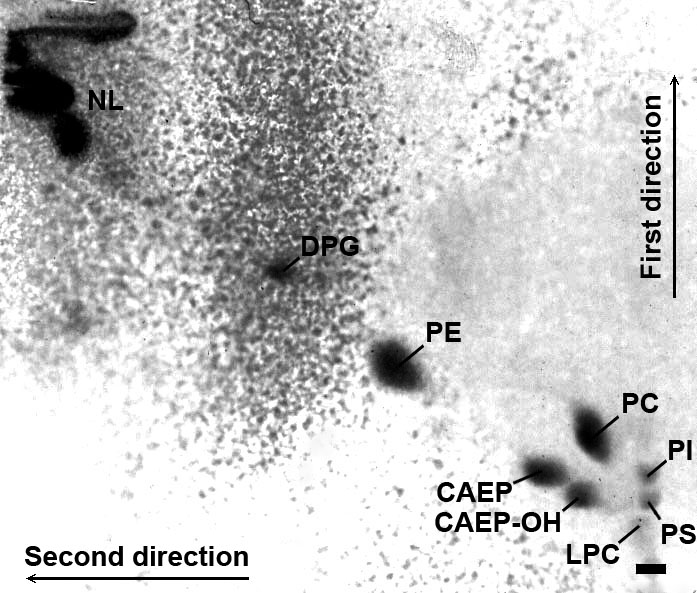


**Figure S2.** Two-dimensional thin layer chromatography of phospholipid classes of *C. lugubris.*

| 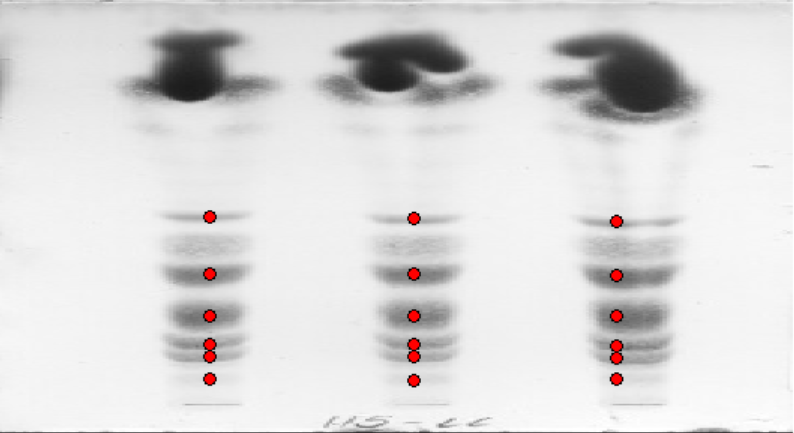  Track 1  Track 2  Track 3  1  2  3  1  2  3  **5**  4  6  **5**  4  6  **5**  4  6  1  2  3 | 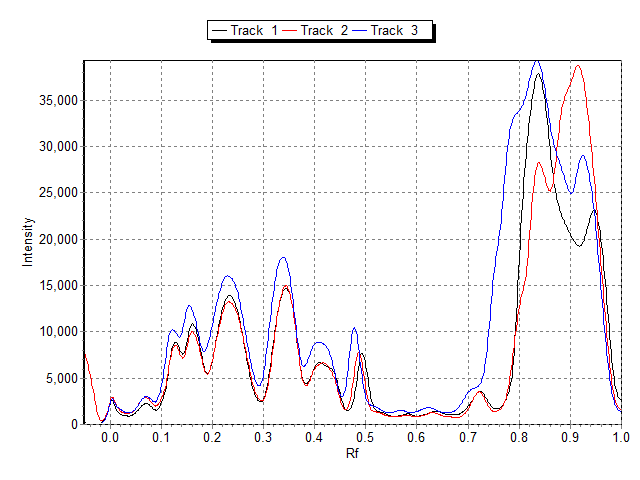  6  5  4  3  1  2 |
| --- | --- |

**Figure S3.** Sorbfil TLC determination of phospholipid classes of the oyster *C. lugubris.* 1 –lysophosphatidylcholine (LPC), 2 – CAEP with hydroxy-FA`s (CAEP-OH) + phosphatidylserine (PS), 3 – ceramide aminoethylphosphonate (CAEP) + phosphatidylinositol (PI), 4 – phosphatidylcholine (PC), 5 – phosphatidylethalnolamine (PE), 6 – diphosphatidylglycerol (DPG).

a)

b)

c)

d)

e)

f)

**Figure S4.** MS and MS^2^ spectra of syhthetic PGA. a), b), c), d) - MS^+^, MS^2+^, MS^-^ and MS^2-^ of 18:0/18:1; e) - MS^2-^ of p18:0/20:4; f) - MS^2-^ of a16:0/18:1 PGA.

a

b

c

| **Figure S5.** Extraced ion chromatograms of synthetic PGA (a) and total lipid extract of *C. lugubris*, (b – intact, c – after mild acid hydrolysis). Peaks at *m/z* 759 and 813 – diacyl, *m/z* 717 – alkyl-acyl, *m/z* 765 and 771 – alkenyl-acyl PGA.  a.  |
| --- |
| b   c  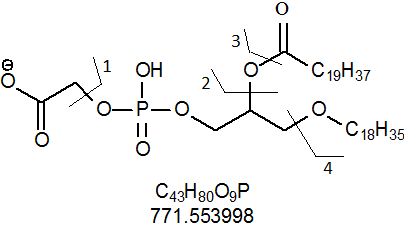 |

**Figure S6.** Fragmentation of PGA 38:1 (p18:0/20:1): a – MS^-^, b – MS^2-^, c – structural formula

a

| b |
| --- |
|  |
| c   |
| d  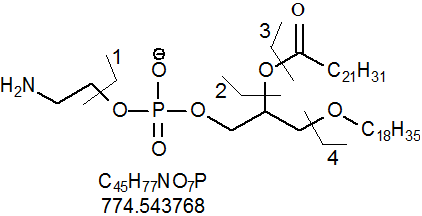 |

**Figure S7.** Fragmentation of PE p40:2 (p18:0/22:2). a – extract ion chromatogram, b – MS^-^, c – MS^2-^, d – fragmentation scheme.

a

|  |
| --- |
| b   |
| c  |
| d  |
| e  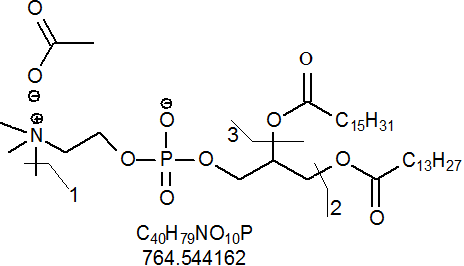 |

**Figure S8.** Fragmentation of PC 30:0 (14:0/16:0). a – extract ion chromatogram; b, c – MS^-^ and MS^2-^ of 764.5452; d – MS^2-^ of 690.5103; e – fragmentation scheme.

| a  |
| --- |
| b  |
| c  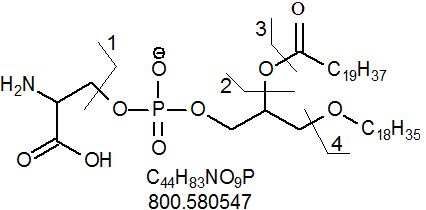 |

**Figure S9.** Fragmentation of PS 38:1 (p18:0/20:1): a – MS^-^; b – MS^2-^; c – fragmentation scheme.

| a  |
| --- |
| b   c  |
| d  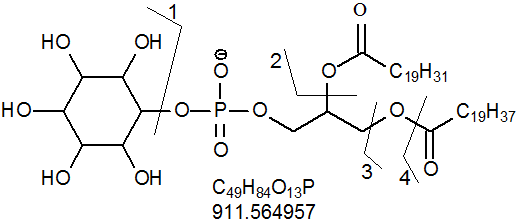 |

**Figure S10.** Fragmentation of PI 40:5 (20:1/20:4): a – MS^-^ b, c – MS^2-^ d – fragmentation scheme.

| a.  |
| --- |
| b.  |

**Figure S11.** Fragmentation of PG 32:0 (16:0/16:0): a – MS^-^; b – MS^2-^.

| a.  |
| --- |
| b.  |

**Figure S12.** Fragmentation of DPG 88:24 (22:6/22:6/22:6/22:6): a – MS^-^; b – MS^2-^.

| a   |
| --- |
| b    c |
|  |

**Figure S123.** HPLC-HRMS of CAEP with Mr=670.: a – Extract ion chromatogram in positive ionization mode; b, c MS-MS – in negative ionization mode at 18.6 min (d20:2/16:0 + d18:2/18:0) and 19.6 min (d19:3/16:0-OH).
